# Intrapatient tacrolimus variability is associated with medical nonadherence among pediatric kidney transplant recipients

**DOI:** 10.3389/frtra.2025.1572928

**Published:** 2025-03-17

**Authors:** Tara B. Gavcovich, Vaka K. Sigurjonsdottir, Marissa J. DeFreitas, Claudia Serrano, Esther Rivas, Migdalia Jorge, Wacharee Seeherunvong, Chryso Katsoufis, Wendy Glaberson, Melisa Oliva, Adela D. Mattiazzi, Carolyn Abitbol, Jayanthi Chandar

**Affiliations:** ^1^ Division of Pediatric Nephrology, Department of Pediatrics, University of Miami Miller School of Medicine, Miami, FL, United States; ^2^ Division of Pediatric Kidney Transplantation, Department of Pediatrics, Miami Transplant Institute, University of Miami Miller School of Medicine, Miami, FL, United States; ^3^ Division of Pediatric Psychology, Department of Psychology, Jackson Health System, Miami, FL, United States; ^4^ Division of Nephrology, Department of Internal Medicine, University of Miami Miller School of Medicine and Jackson Memorial Hospital, Miami Transplant Institute, Miami, FL, United States

**Keywords:** kidney transplant, pediatric, adherence, tacrolimus variability, tacrolimus, pediatric kidney transplant recipients, medication nonadherence

## Abstract

**Background:**

Long-term survival of kidney allografts is limited by multiple factors, including nonadherence. High intrapatient variability in tacrolimus levels (≥30%) is associated with *de novo* donor-specific antibody (*dn*DSA) formation, increased risk of rejection and graft loss.

**Methods:**

We prospectively analyzed the association between tacrolimus intrapatient variability and nonadherence in pediatric kidney transplant recipients. We derived a composite adherence score from 0 to 3 points based on (1) Basel Assessment of Adherence to Immunosuppressive Medical Scale^©^; (2) healthcare team score; and (3) intentionally missed laboratory or clinic visits. A score of 1 or more was considered nonadherent. Tacrolimus 12 h trough levels, patient characteristics and clinical outcomes were collected. Tacrolimus IPV was calculated as the coefficient of variation.

**Results:**

The nonadherent group had a significantly higher median tacrolimus intrapatient variability (31%) as compared to the adherent cohort (20%) (*p* < 0.001.) Tac IPV demonstrated strong predictive performance for adherence (AUC 0.772), with a particularly high sensitivity of 90% at thresholds up to 20%, offering a practical and actionable framework for assessing adherence-related risks in clinical practice.

**Conclusions:**

Tacrolimus intrapatient variability may be a useful biomarker to identify nonadherence and high-risk patients, allowing for early interventions to prevent adverse graft outcomes.

## 1 Introduction

Allograft rejection due to insufficient immunosuppression is a major contributor to early graft loss (1–3). Not taking medications consistently or inconsistent follow-up with the medical care team is a known risk factor for poor graft outcomes, with nonadherence being the most common in adolescent patients (1, 4, 5). Post-transplant maintenance immunosuppression typically includes tacrolimus, a calcineurin inhibitor, with a narrow therapeutic index requiring frequent drug level monitoring to balance effective drug concentrations while minimizing toxicity (6–8). High tacrolimus intrapatient variability (Tac IPV) has been studied for over two decades and measured with two different methods, the coefficient of variation (CV) and Medication Level Variability Index (MLVI). Tac IPV has been increasingly recognized as a biomarker for graft rejection and loss (7, 9–12) and a marker of nonadherence (13). The Medication Adherence in children who had a Liver Transplant (MALT) prospective multi-site study evaluated whether MLVI predicts late acute rejection. A total of 379 participants were followed prospectively and results showed that a higher prerejection MLVI predicted adverse graft outcomes (14).

The International Consensus on Managing Modifiable Risk in Transplantation recommends monitoring nonadherence as a fifth vital sign (15). As expected, nonadherence has also been associated with worse graft outcomes, leading to increased incidence of rejection, *de novo* donor specific antibody (*dn*DSA) formation, decreased renal function and ultimately graft loss (1, 16, 17). While multiple factors contribute to raising the Tac IPV, including tube feeding, feeding intolerance, infection, drug or food interactions and dose adjustments (6, 8, 12, 18–21), medication nonadherence is thought to be the strongest contributor (22). Several studies have shown that tacrolimus variability responds to behavioral interventions, strongly supporting that is affected by behavior (23–26). This relationship, however, has not been described well in adults or children, especially since nonadherence is difficult to measure consistently in the clinical setting (27, 28). The prevalence of nonadherence varies across studies, and depends on heterogenous measurement tools, which adds to the inconsistent analyses (29). Measures of nonadherence include direct (observation, drug assays) and indirect (self-report, collateral report, prescription refills, electronic monitoring) parameters, and there is no single ideal method given the limitations of each (27, 29–31).

Since adherence measures can be quite unreliable and burdensome to patients and clinicians, tacrolimus variability has been suggested as a potential objective biomarker for nonadherence, specifically in pediatric liver transplant patients (14). We hypothesized that nonadherence was a strong contributor to high Tac IPV. The aim of this study was to investigate the relationship between Tac IPV and adherence in a cohort of children and young adult kidney transplant recipients.

## 2 Methods

### 2.1 Study design and population

This is a prospective, single center, cross-sectional study. This research was approved by the institutional review board of the University of Miami Miller School of Medicine (IRB #20220914). All participants provided informed consent. All pediatric recipients of isolated kidney transplants who presented to Miami Transplant Institute (MTI) for a post-transplant visit from September 2022 to December 2022 were considered eligible for inclusion in the study. Patients < 10 months post-transplant, not on tacrolimus immunosuppression, or with fewer than three tacrolimus levels in the study period were excluded. The standard induction protocol included thymoglobulin on post-transplant day 0 (one dose total of thymoglobulin), basiliximab on post-transplant day 0 and post-transplant day 3 or 4 (two doses total of basiliximab), along with a steroid taper. Maintenance immunosuppression included tacrolimus and mycophenolate mofetil, with or without prednisone, based on immunologic risk. Sirolimus was selectively added for some recipients to diminish target tacrolimus levels and to limit nephrotoxicity. The goal tacrolimus (or combined tacrolimus and sirolimus) level was 6–8 in the first three months post-transplant and 5–7 thereafter. Adherence data was collected on the day of enrollment during the clinic visit. Baseline and follow-up laboratory data was collected prospectively through November 2023. Historical data on episodes of rejection and *dn*DSA formation was also collected. All participants were followed for at least 6 months after enrollment. Demographic and clinical characteristics such as sex, age at transplantation, duration after transplant, underlying renal disease, donor source, pre-transplant panel reactive antibody (PRA), human leukocyte antigen (HLA) matching, and insurance type were obtained from the electronic medical record (EMR).

### 2.2 Composite adherence score

In addition to the validated Basel Assessment of Adherence to Immunosuppressive Medical Scale^©^(BAASIS^©^), we developed a composite adherence score (CAS) which included the BAASIS^©^ to enhance our assessment of adherence and address limitations of self-report, which are known to include biases such as recall inaccuracies and social desirability bias, leading to underreporting of nonadherence. We used a CAS ranging from 0 to 3 total points based on three parameters: (1) Basel Assessment of Adherence to Immunosuppressive Medical Scale^©^(BAASIS^©^); (2) healthcare team score; and (3) intentionally missed laboratory or clinic visits. Each measure was awarded 1 point if considered nonadherent. The final CAS score was 0 to 3. A perfect adherence score corresponded to a total score of 0, and nonadherence was defined as a score of 1–3.

#### 2.2.1 BAASIS^©^

The BAASIS^©^ is a written questionnaire that is widely used in research and clinical practice, and has been validated in kidney transplant recipients to assess adherence to immunosuppressive medications (32–34). It consists of five questions on timing and taking of immunosuppressive medications, including missed doses, drug holidays, time deviation, and dose changes or discontinuation of the medications without physician consultation. The questionnaire was filled out independently by the patient (if ≥15 years old) or caregiver (if younger) at the time of enrollment, based on a 4-week recall. Nonadherence was defined as “yes” to any of the questions.

#### 2.2.2 Care team score

The transplant clinical team (three physicians, two nurse coordinators, and one nurse practitioner closely involved in the follow-up care of the kidney transplant recipients) scored recipients’ adherence on 4-point scale (poor, suboptimal, fair, good), as described by Schafer et al. (27). A patient received a score of 4 if all clinicians estimated his/her adherence as good, a score of 2 or 3 if any of the providers estimated his/her adherence as less than good (fair or suboptimal), but not poor, and a score of 1 if any clinician estimated his/her adherence as poor, independently of the estimations given by the other clinicians. A perfect adherence score corresponded to a total score of 4, and nonadherence was defined as a score of 1–3.

#### 2.2.3 Intentionally missed laboratory or clinic visits

Transplant nurse coordinators track missed clinic and laboratory visits as a standard. Nonadherence was defined as report of more than one intentionally missed clinic and/or laboratory visit. An intentionally missed visit was defined as patient and/or caregiver not providing an explanation for missing the visit, not trying to reschedule the visit, and/or not calling the healthcare team prior to missing the visit.

### 2.3 Tacrolimus intrapatient variability

We collected all available 12 h trough tacrolimus levels in the six to twelve-month period following enrollment. Levels drawn while hospitalized, during sickness, or non-trough levels were excluded. Tac IPV was calculated using the CV according to the equation CV = σ/μ × 100%, where *σ* is the standard deviation of the tacrolimus levels and μ is the mean tacrolimus level.

### 2.4 Statistical analysis

Baseline characteristics and demographics were summarized descriptively using median (interquartile ranges) and counts (percentages) where appropriate. Receiver operating characteristic (ROC) curves were generated to evaluate diagnostic performance, and the area under the curve (AUC) was calculated. A *p* value < 0.05 was considered statistically significant. Analyses were performed using GraphPad Prism 10.0 software.

## 3 Results

### 3.1 Study population

From September 2022 through December 2022, 75 patients were enrolled and followed through November 2023, with a total of 725 tacrolimus levels (median, 9 levels per patient; interquartile range 6,13) analyzed. Twelve patients did not meet inclusion criteria. No eligible patient refused to participate. The median follow-up time was 12 months (IQR 10,12). Median age at transplant was 14 years (IQR 7.5,16.5). The median post-transplant time at enrollment was 3.1 years (IQR 1.5,15.2). Demographic characteristics, stratified by adherence as defined by CAS, are summarized in Table 1. In addition to tacrolimus, 28% of participants were also on sirolimus (19 patients) or abatacept (2 patients). All participants continued tacrolimus throughout the study period.

**Table 1:** Comparison of adherent and nonadherent patients as defined by CAS (*n* = 75).

Characteristic	Adherent	Nonadherent	*p*-value
*N* (%)	38 (51)	37 (49)	
Age at enrollment (years), median [IQR]	17 [12,19]	16 [13,20]	0.84
Sex, *n* (%)			
Female, *n* (%)	10 (26)	14 (38)	0.33
Male, *n* (%)	28 (74)	23 (62)	
Race, Ethnicity, *n* (%)			
Black, Non-Hispanic, *n* (%)	10 (26)	17 (46)	0.21
Black, Hispanic, *n* (%)	0 (0)	1 (3)	
White, Non-Hispanic, *n* (%)	4 (11)	3 (8)	
White, Hispanic, *n* (%)	22 (58)	16 (43)	
Asian, Non-Hispanic, *n* (%)	2 (5)	0 (0)	
Time since transplant (years), median [IQR]	3 [1,5]	4 [2,6]	0.31
Insurance type, *n* (%)			
Medicaid, *n* (%)	16 (42)	23 (62)	0.18
Medicare, *n* (%)	12 (32)	6 (16)	
Private Insurance, *n* (%)	10 (26)	8 (22)	
History of TCMR, *n* (%)	4 (10)	17 (46)	<0.001
History of ABMR, *n* (%)	1 (3)	5 (10)	0.11
Proteinuria, *n* (%)	3 (8)	6 (16)	0.31
History of *dn*DSA Class II, *n* (%)	16 (42)	25 (68)	<0.001
IPV (%), median [IQR]	20 [15,26]	31 [23,43]	<0.001
CAS, median [IQR]	0 [0]	2 [1,4]	<0.001

IPV, intrapatient variability; TCMR, T-cell mediated rejection; ABMR, antibody-mediated rejection; proteinuria, urine protein/creatinine ratio above 0.5 mg/mg that persisted over 3 months; *dn*DSA, *de novo* donor-specific antibodies; CAS, composite adherence score.

### 3.2 Tacrolimus intrapatient variability and adherence

The median Tac IPV among all participants was 24% (IQR 17,33). Among participants who were not on sirolimus or abatacept, the median tacrolimus level was 5.5 ng/ml (IQR 4.5,6.7). Among patients on concomitant sirolimus or abatacept, and therefore with lower tacrolimus goals, the median tacrolimus level was 3.2 ng/ml (IQR 2.5,4.3).

**Figure 1:**
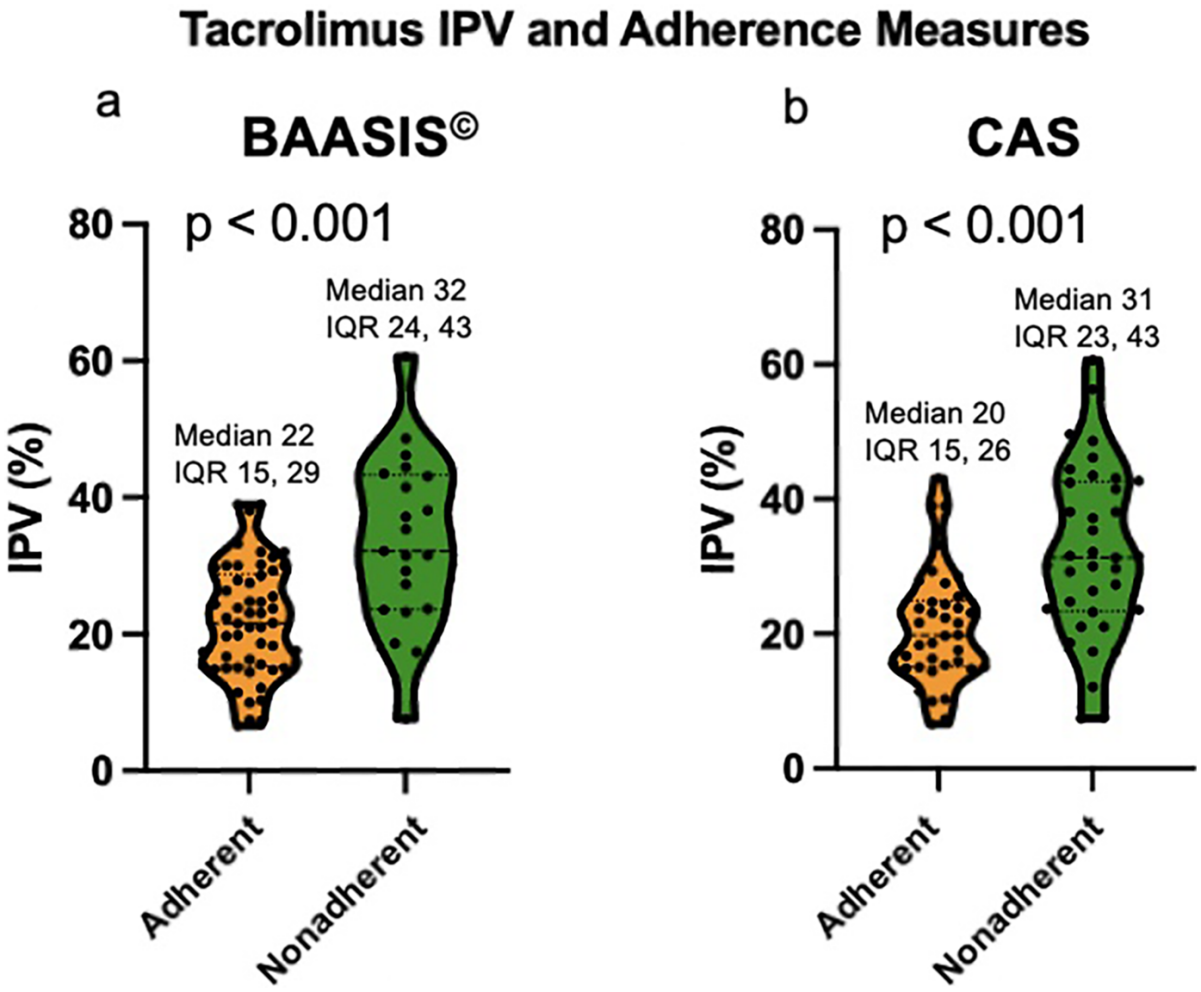
**(a)** Violin plot of Tac IPV comparing adherent and nonadherent patients based on BAASIS^©^. **(b)** Violin plot of Tac IPV comparing adherent and nonadherent patients based on CAS. IPV: intrapatient variability; BAASIS©: Basel Assessment of Adherence to Immunosuppressive Medical Scale; CAS: composite adherence score.

Using the BAASIS^©^ alone, the nonadherence rate was 29%; the nonadherent group had a median Tac IPV of 32%, vs. 22% among the adherent cohort (*p* < 0.001, Figure 1a).

**Figure 2:**
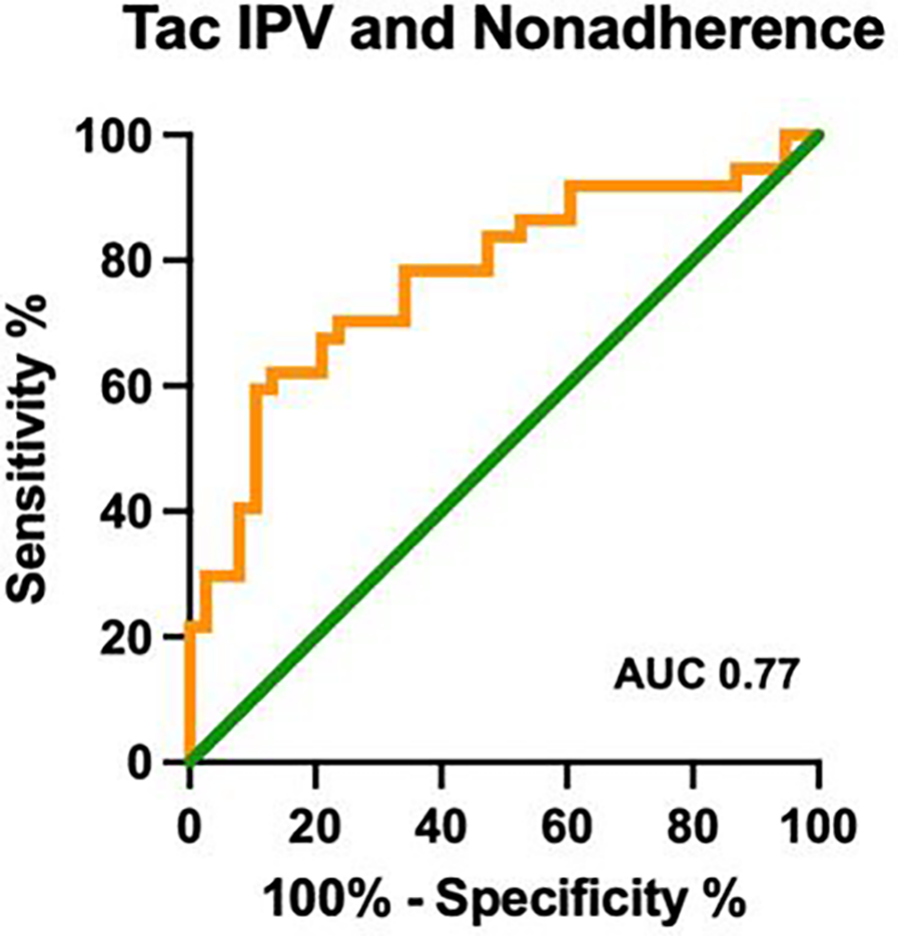
ROC curve of Tac IPV in identifying nonadherence. ROC: receiver operating characteristic; Tac IPV: tacrolimus intrapatient variability; AUC: area under the curve; CAS: composite adherence score.

Using the CAS, the nonadherence rate was 49%; the nonadherent group had a significantly higher median Tac IPV of 31%, as compared to the adherent cohort with a median Tac IPV of 20% (*p* < 0.001, Figure 1b). Table 1 compares the clinical and demographic characteristics between patients classified as adherent vs. nonadherent. The performance of the Tac IPV in predicting nonadherence in pediatric kidney transplant recipients was assessed using an ROC curve. The AUC was 0.77 (95% CI, 0.66 to 0.88), indicating a moderately strong ability to distinguish between adherent and nonadherent patients. To stratify patients into risk categories, we analyzed Tac IPV thresholds along the ROC curve. The optimal cut-off point was a Tac IPV of 17%, where sensitivity reached 92%, suggesting that IPV values below this threshold were highly associated with adherence (Figure 2). Generally, patients with a Tac IPV 20% or less could be categorized as low risk, with high sensitivity at 90% and specificity at 60% (Figure 2). ROC curve based on BAASIS^©^ alone had an AUC of 0.74 (95% CI, 0.61 to 0.86).

## 4 Discussion

This prospective study highlights the importance of tacrolimus intrapatient variability as a biomarker for identifying nonadherence in pediatric kidney transplant recipients. Our findings demonstrate a significant association between high Tac IPV and nonadherence, supporting its potential as a reliable, objective tool for detecting patients at increased risk of adverse outcomes. These results are consistent with prior studies across both pediatric and adult transplant populations, which have demonstrated that increased Tac IPV is associated with higher rates of rejection and graft loss (1, 4, 5, 35, 36).

In our study, median Tac IPV in the entire cohort was 24%, lower than studies that did not censor out data (12), and higher than other studies assessing highly adherent patients (12, 26, 27, 37). Median Tac IPV was lower in adherent patients, 20% vs. 31% in nonadherent. In the MALT study, MLVI correlates strongly with adherence measures, including electronic monitoring and multidisciplinary panel evaluations, reinforcing its role as a behavioral metric rather than a reflection of pharmacologic variability (14). The MALT study further demonstrated that the MLVI is not influenced by metabolic or absorption anomalies, common drug-drug interactions, or prescription practices.

Additionally, the MLVI is best utilized as a threshold construct rather than a continuous measure, consistent with our findings from the ROC analysis. Tac IPV exhibited strong predictive performance for adherence, with an AUC of 0.77, supporting its utility as a robust marker in clinical practice. Notably, Tac IPV was particularly sensitive at thresholds up to 20% for identifying adherent patients, providing a practical and clinically actionable framework for assessing adherence-related risks.

The use of a threshold-based approach offers significant advantages in risk stratification and intervention planning. Specifically, Tac IPV exceeding 30% should prompt heightened clinical suspicion for nonadherence, warranting further evaluation and direct discussions to uncover and address potential barriers to adherence. This practical application allows for early identification of at-risk patients and facilitates targeted interventions that may improve both adherence and clinical outcomes. These findings reinforce the utility of variability indices such as MLVI and Tac IPV as objective tools to improve risk stratification and optimize clinical care.

Leino et al. evaluated baseline patterns of Tac IPV in an adherent cohort of adult kidney and liver transplant recipients. The study population demonstrated 99.9% adherence, as measured by patient daily diary, pill counts and the electronic medication event monitoring system (MEMS); the median weekly Tac IPV was calculated at 15.2% (37). This finding indirectly suggests that tacrolimus levels are not variable in adherent patients. In a *post-hoc* analysis of a dataset from a randomized controlled trial, Ko et al. looked at the relationship between adherence, as measured by self-report and MEMS, and Tac IPV in adult kidney transplant recipients. The median Tac IPV was not significantly different between adherent and nonadherent groups, 16 vs. 16.5% (26). This was concordant with a paper by Gokoel et al. that also showed a lower mean Tac IPV of 17.9% and no relationship between adherence and Tac IPV among stable adult kidney transplant recipients (25). The baseline Tac IPV in our cohort was higher than in these recent adult studies, suggesting an underlying difference (25, 26, 37). The baseline Tac IPV in a recent pediatric study from Piburn, et al. was 30%, higher than in our study, which is likely explained by their retrospective design as well as inclusion of all uncensored trough levels (12).

A highly variable drug level has been defined in many studies as ≥30%, but in the two studies by Ko et al. and Gokoel et al., median Tac IPV was low, probably because the degree of nonadherence was not sufficient in these cohorts to test the hypothesis (25, 26). Given the difficulty to engage nonadherent patients in research, trials are often biased towards a sample of adherent patients as supported by a recent systematic analysis (28). In the study by Ko et al., the cohort consisted of motivated patients that participated in a randomized controlled trial, with a mean age at transplant of 43 years (26). In our cohort, almost half of the patients were nonadherent at a median age of 17 years. It is well known that recipients aged 14 to 16 years have the greatest risk of kidney graft failure (1, 5, 16, 38). As described by Piburn et al., the baseline trend of Tac IPV started to increase in adolescence and young adulthood, which could indicate an increased incidence of nonadherence by this age group (12). In our cohort, nonadherent patients had a higher Tac IPV and were more likely to have a history of biopsy-proven rejection and formation of *dn*DSAs, suggesting that nonadherent behavior probably preceded our assessment. We identified an association between adherence and Tac IPV, where the nonadherent group demonstrated a high-risk Tac IPV of ≥30%, which had not been previously done prospectively.

One of the largest strengths of this study was the prospective study design, and the real time collection of data, allowing us to limit confounders such as improper timed levels, levels drawn during hospitalization or comorbid illness, or a change in therapeutic goal during acute infection or graft rejection. Limitations include the lack of objective measures of adherence. This shows the difficulty of truly assessing adherence in the clinical setting and in research. While BAASIS^©^ is a validated tool for assessing adherence, it is limited by self-report bias, which can result in underestimation of nonadherence. Self-reported adherence, such as BAASIS^©^, has known biases including social desirability bias and recall inaccuracies, leading to underreporting of nonadherence. Additionally, nonadherent patients are less likely to participate in studies, introducing selection bias or the streetlight effect, where adherence is assessed primarily in the most accessible patients. To enhance the accuracy of adherence assessment and mitigate the limitations of self-report, we developed the CAS, incorporating more objective markers such as intentionally missed clinic/laboratory visits and healthcare team assessments, providing a more robust and clinically relevant evaluation of adherence.

The composite score has not yet been validated, which limits our study; however, the BAASIS^©^ is a validated measure used among kidney transplant recipients to assess adherence to immunosuppressive medications and remains a component of our CAS (32–34, 39). Further, BAASIS^©^ alone demonstrated sufficient statistical power in detecting the correlation between adherence and Tac IPV, as evidenced by the strong relationship observed in our analysis. However, the CAS provided greater sensitivity in identifying Tac IPV, our surrogate marker of nonadherence, reinforcing the importance of multi-method adherence assessment. The study was not blinded; therefore, the providers scoring for nonadherence were also caring for the patients, allowing them to make informed assessments of adherence, but also allowing for a possibility of bias. Our study was further limited by being from a single center and having a relatively short follow-up time.

To conclude, this study demonstrates the important role of Tac IPV as a biomarker for identifying nonadherence and predicting adverse graft outcomes in pediatric kidney transplant recipients. Tac IPV was significantly higher in nonadherent patients and the low risk threshold for Tac IPV was identified to be less than 20%. Adolescents, a population particularly vulnerable to lapses in adherence, present unique challenges that require targeted strategies to ensure consistent immunosuppression. Tac IPV offers an objective and noninvasive metric to identify at-risk patients early, enabling timely interventions to mitigate the risk of acute rejection and graft loss. Furthermore, the potential modifiability of Tac IPV through adherence-promoting interventions highlights its clinical relevance not only as a diagnostic tool but also as a target for improving long-term outcomes.

The long-term care of pediatric kidney transplant recipients necessitates a multidisciplinary approach, with adherence monitoring at its core. Tac IPV serves as a valuable adjunct in this paradigm, bridging the gap between subjective assessments and actionable insights. Future multicenter, prospective studies are essential to validate Tac IPV as a reliable biomarker and to explore its role in guiding personalized interventions. By integrating Tac IPV monitoring into routine clinical practice, clinicians can better address the complexities of nonadherence, ultimately improving outcomes for pediatric and adolescent kidney transplant recipients.

## Data Availability

The raw data supporting the conclusions of this article will be made available by the authors, without undue reservation.
