# Supplementary material for: Intrapatient tacrolimus variability is associated with medical nonadherence among pediatric kidney transplant recipients
Source: Front Transplant. 2025 Mar 17;4:1572928. doi: 10.3389/frtra.2025.1572928 (PMC11955662; doi:10.3389/frtra.2025.1572928)
Supplement: Supplementary file 1 [file Table1.docx]

**SUPPLEMENTARY TABLE.** Adherence Measures

| **Adherence Measure** | **Definition of Nonadherence** | **Point Towards CAS** |
| --- | --- | --- |
| BAASIS^©^ | - Missed one or more doses in the last 4 weeks - Took all prescribed doses, but had a time deviation >2 h from prescribed time in the last 4 weeks - Changed dose of medication or stopped medication without clinician prescription in the last 4 weeks | 1 |
| Care Team Score | - Score of 1-3 (poor, suboptimal, fair) based on transplant clinician care team estimations of adherence | 1 |
| Missed clinic or laboratory visits | - More than one intentionally missed visit | 1 |
| COMPOSITE ADHERENCE SCORE | Score of 1-3 | Maximum total possible: 3  Minimum total possible: 0 |

BAASIS^©^: Basel Assessment of Adherence to Immunosuppressive Medical Scale; CAS: composite adherence score
